# Exploring mechanisms of ventricular enlargement in idiopathic normal pressure hydrocephalus: a role of cerebrospinal fluid dynamics and motile cilia

**DOI:** 10.1186/s12987-021-00243-6

**Published:** 2021-04-19

**Authors:** Shigeki Yamada, Masatsune Ishikawa, Kazuhiko Nozaki

**Affiliations:** 1 Department of Neurosurgery, Shiga University of Medical Science, Seta Tsukinowa-cho, Otsu, Shiga, 520-2192 Japan; 2 Department of Neurosurgery and Normal Pressure Hydrocephalus Center, Rakuwakai Otowa Hospital, Kyoto, Japan; 3 Interfaculty Initiative in Information Studies, Institute of Industrial Science, The University of Tokyo, Tokyo, Japan; 4 Rakuwa Villa Ilios, Kyoto, Japan

**Keywords:** Ventriculomegaly, Idiopathic normal pressure hydrocephalus, Fluid dynamics, Oscillatory shear stress, Motile cilia, Ependymal cell

## Abstract

Idiopathic normal pressure hydrocephalus (iNPH) is considered an age-dependent chronic communicating hydrocephalus associated with cerebrospinal fluid (CSF) malabsorption; however, the aetiology of ventricular enlargement in iNPH has not yet been elucidated. There is accumulating evidence that support the hypothesis that various alterations in CSF dynamics contribute to ventricle dilatation in iNPH. This review focuses on CSF dynamics associated with ventriculomegaly and summarises the current literature based on three potential aetiology factors: genetic, environmental and hydrodynamic. The majority of gene mutations that cause communicating hydrocephalus were associated with an abnormal structure or dysfunction of motile cilia on the ventricular ependymal cells. Aging, alcohol consumption, sleep apnoea, diabetes and hypertension are candidates for the risk of developing iNPH, although there is no prospective cohort study to investigate the risk factors for iNPH. Alcohol intake may be associated with the dysfunction of ependymal cilia and sustained high CSF sugar concentration due to uncontrolled diabetes increases the fluid viscosity which in turn increases the shear stress on the ventricular wall surface. Sleep apnoea, diabetes and hypertension are known to be associated with the impairment of CSF and interstitial fluid exchange. Oscillatory shear stress to the ventricle wall surfaces is considerably increased by reciprocating bidirectional CSF movements in iNPH. Increased oscillatory shear stress impedes normal cilia beating, leading to motile cilia shedding from the ependymal cells. At the lack of ciliary protection, the ventricular wall is directly exposed to increased oscillatory shear stress. Additionally, increased oscillatory shear stress may be involved in activating the flow-mediated dilation signalling of the ventricular wall. In conclusion, as the CSF stroke volume at the cerebral aqueduct increases, the oscillatory shear stress increases, promoting motor cilia shedding and loss of ependymal cell coverage. These are considered to be the leading causes of ventricular enlargement in iNPH.

## Introduction

**Fig. 1:**
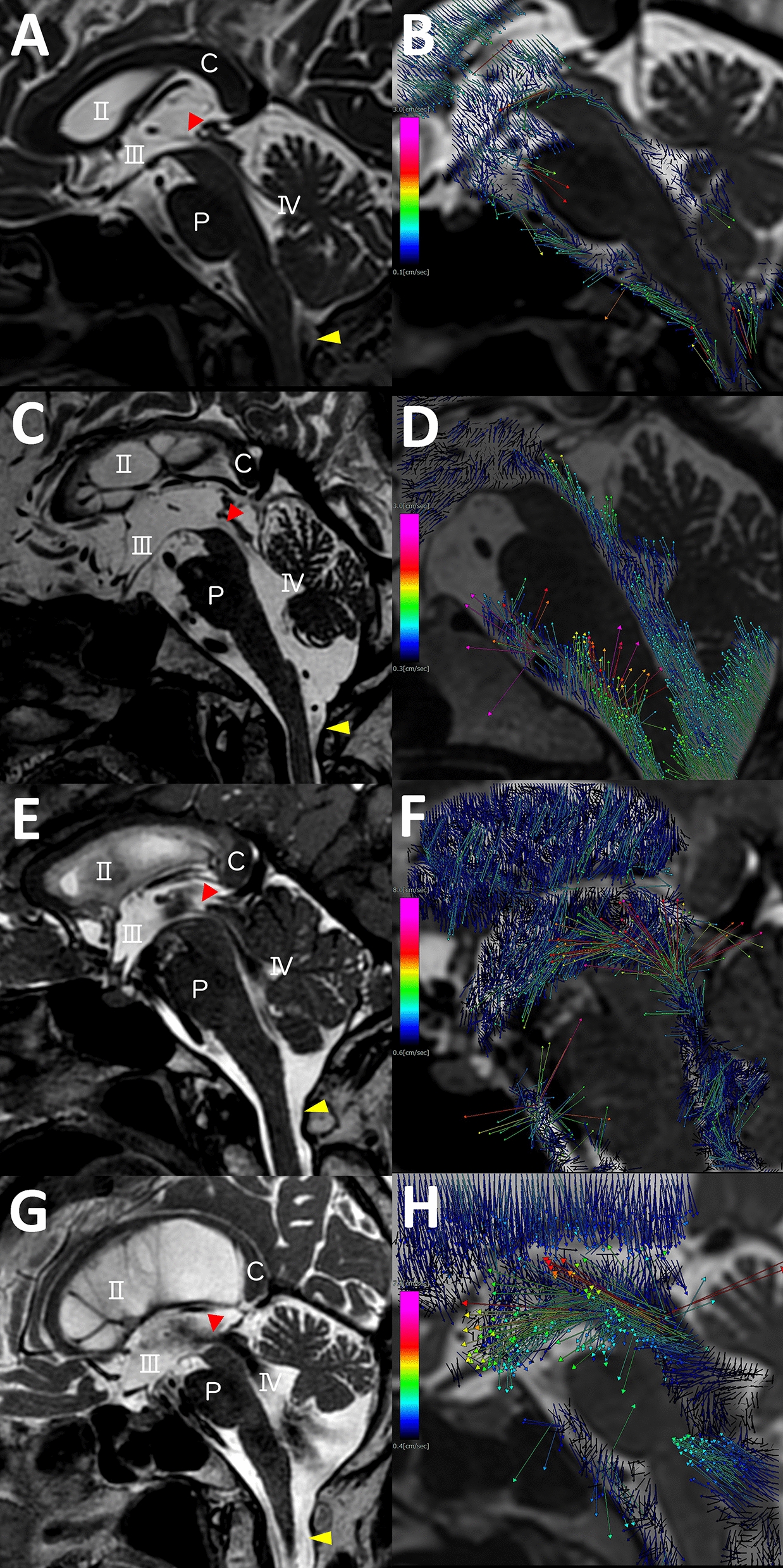
Flow void signs and flow vectors in normal elderly and three types of normal-pressure hydrocephalus (NPH). The left figures (**a**, **c**, **e**, **g**) depict the flow void sign at the top of the cerebral aqueduct (red arrow head) and dorsal region of the foramen magnum (yellow arrow head) from the T2-weighted fast spin-echo sequence, and the right figures (**b**, **d**, **f**, **h**) show the flow vectors from 4D flow MRI in normal elderly (**a** and **b**) and patients diagnosed with idiopathic NPH (**c** and **d**), secondary NPH (**e** and **f**) and late-onset congenital or developmental hydrocephalus (**g** and **h**). The colour of the vector represents flow velocity; pink indicates highest velocity and blue indicates lowest velocity. C: corpus callosum, P: pons, II: lateral ventricle, III: third ventricle, IV: fourth ventricle

Hydrocephalus is characterised by pathological enlargement of the ventricles. Communicating chronic hydrocephalus in adult is synonymous with normal pressure hydrocephalus (NPH) in which the ventricles expand while the intracranial pressure remains within normal range [1]. Patients with NPH present the typical triad of cognitive decline, gait imbalance and urinary incontinence. NPH has been categorised into congenital/developmental aetiologies, idiopathic (iNPH) and secondary NPH (sNPH) developing after haemorrhagic stroke, trauma, infection and tumour [2–6]. The pathophysiology of iNPH has not yet been elucidated, although advanced age and alterations in cerebrospinal fluid (CSF) dynamics have been recognised as potential contributory factors. Initially, iNPH was considered as a disease associated with CSF malabsorption, due to the factor that the intracranial CSF volume in iNPH was significantly larger than that in age-matched controls or patients with other brain diseases [7]. Typical iNPH neuroimaging findings were expressed as disproportionally enlarged subarachnoid space hydrocephalus (DESH), characterised by ventricular enlargement, high convexity/midline tight sulci, and enlarged Sylvian fissures and basal cistern [5–9]. As an aetiology of DESH-pattern CSF distribution in iNPH, we previously reported that the inferior horn of the lateral ventricles directly communicates with the basal cistern via the choroidal fissure, serving as an overflow device that works with increased volume of intracranial CSF [5]. However, the mechanisms contributing to the enlargement of ventricles rather than subarachnoid spaces in iNPH remain to be determined. The development of imaging technology has enabled the observation of pulsatile and bidirectional CSF movements that are mainly driven by blood circulation and respiration under normal conditions [10–18]. CSF normally flows outward from the cranium during systole due to the expansion of the brain and intracranial vasculature, and flows inward during diastole. The CSF stroke, corresponding to one reciprocating motion during a cardiac cycle, is known to be increased at the cerebral aqueduct in most NPH patients [10–14, 16–18]. Similarly, the flow void sign, determined by signal loss on T2-weighted fast or turbo spin echo sequence that indicates a fast flow velocity, as depicted in Fig. 1, was also reported to be prominent at the cerebral aqueduct in NPH patients [11, 12, 19]. Previously, we found that an increased CSF stroke volume at the lower end of the cerebral aqueduct in iNPH was significantly associated with the dilation of the foramen of Magendie [17]. Therefore, we hypothesised that the foramen of Magendie serves as the first water gate to reduce backward CSF inflow into the ventricular system, and its subsequent dilation leads to an increase reverse flow into the ventricle systems. Furthermore, an increase in CSF stroke volume at the foramen of Monro, and not at the cerebral aqueduct, was significantly associated with the dilation of the lateral ventricles toward the vertex in iNPH [17]. Recently, CSF flow produced by the directional beating of the motile cilia on the ventricle surfaces was reported to be crucial in the regulation of the CSF distribution within and across brain ventricles, and the dysfunction of ciliary beat leads to reduced hydrodynamic coupling between the ventricles [20]. Additionally, coordinated directional beating of ependymal cilia is essential for appropriate CSF flow [21–23]. Therefore, motile cilia lining ventricular ependymal surfaces can be considered as a mechanism of ventricular enlargement in the association with CSF stroke volume. Thus, we speculated that the coordinated directional movements of the motile cilia on the apical surface of ependymal cells may be impaired by an increase in CSF stroke volume. To investigate a possible mechanism for the relationship between the development of ventricular enlargement and degeneration of ependymal cilia in iNPH, we reviewed the current literature on genetic and environmental factors for ventricular enlargement in communicating hydrocephalus.

### Current concept of CSF dynamics

**Fig. 2:**
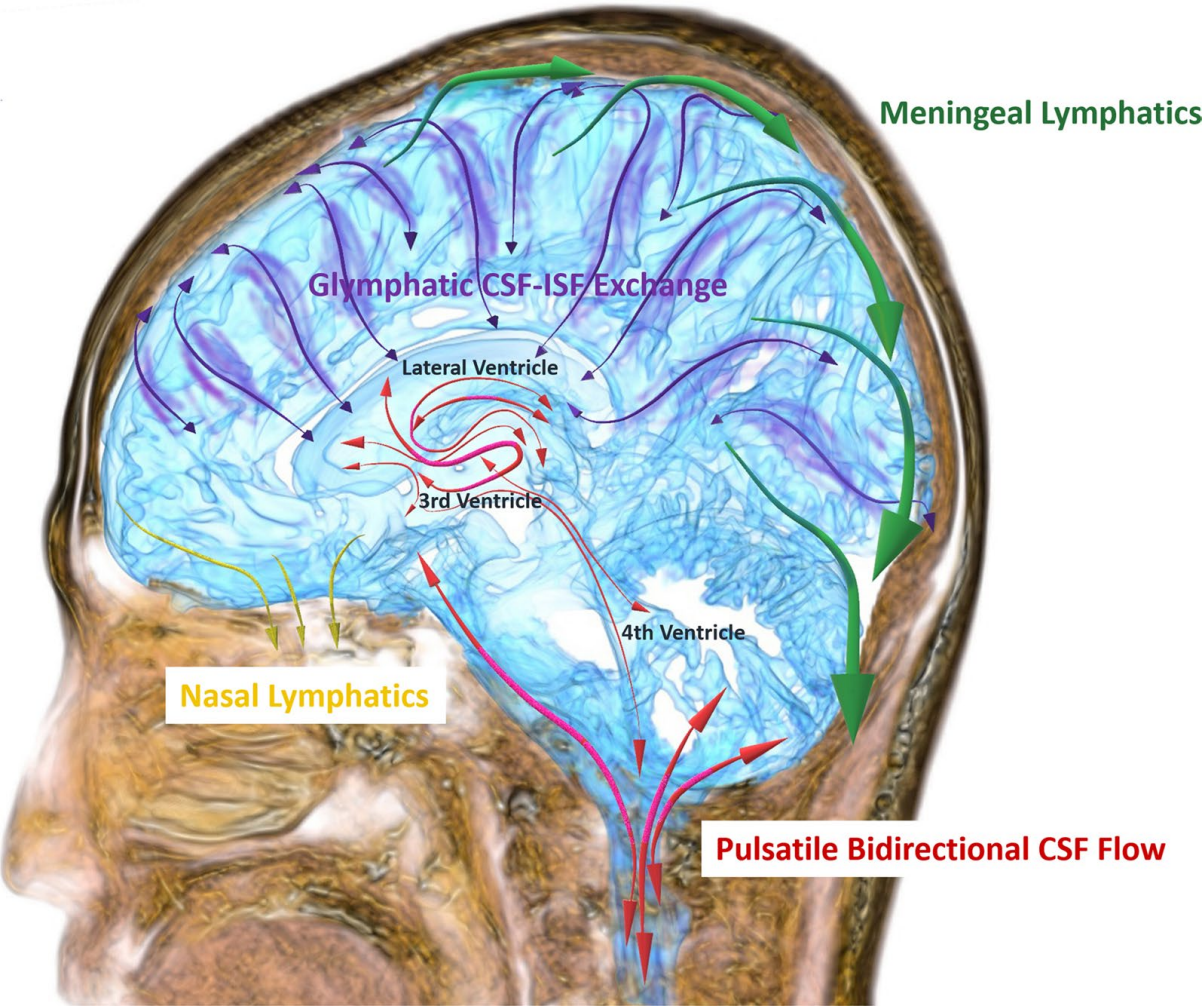
Current concept of CSF dynamics. This figure was drawn by using the Paint 3D application (Microsoft Corporation) and SYNAPSE 3D (FUJIFILM Corporation). The colour arrows indicate direction of CSF movement

In the early 1900s, Cushing proposed the third circulation theory that CSF is secreted by the choroid plexus in the ventricles, flows from the lateral ventricles to the third and fourth ventricles via the foramina of Monro and cerebral aqueduct, moves to the subarachnoid spaces through the foramina of Magendie and Luschka, circulates from the basal cistern to the subarachnoid spaces in the convexity part of the brain, and finally be absorbed into the blood through the arachnoid villi [24]. Although many researchers continue to believe third circulation by the CSF bulk flow, remarkable research findings of the last decade have questioned this traditional understanding of CSF physiology [25–33]. The current concept of CSF dynamics was summarized in Fig. 2. First, magnetic resonance flow images visualized complex pulsatile CSF motion (red double-headed arrows in Fig. 2), not bulk flow [10–18]. Second, in the glymphatic pathway, interstitial fluid flows from the arterial perivascular space to the venous perivascular space in the brain parenchyma and exchanges to the CSF through the water channel aquaporin-4 (purple double-headed arrows) [26, 27, 30, 31]. Therefore, the efflux of interstitial fluid can be the main CSF production, rather than the choroid plexus. Finally, CSF drains from the subarachnoid spaces to the nasal lymphatics via the cribriform plate (yellow arrows) [33] and to the meningeal lymphatics (green arrows) [26, 28, 29, 32] These lymphatic drainages were considered as the major outflow pathway rather than the dural venous sinuses through arachnoid villi. Based on these new concepts of CSF dynamics, we considered the mechanisms of ventricular enlargement in communicating hydrocephalus.

### Genetic factors

Genetic-based pathways offer valuable insights into understanding the underlying mechanisms of communicating hydrocephalus. There has been accumulating evidence which supports the association of ciliary dysfunction lining the ventricle surfaces with congenital hydrocephalus [22, 34–36]. Several mutations in *Ccdc39*, *Celsr2*, *Celsr3*, *Cetn2*, *Dvls*, *FoxJ1*, *Hydin*, *Mdnah5*, *Pkd1*, *Tg737, Daple, Dnah14, Cfap43 and Cwh43* genes, which were related to the structure or function of motile cilia on ependymal cells, have been reported to develop hydrocephalus in animal models [21, 22, 34, 37–51]. For example, the transcription factor *FoxJ1* plays a crucial role in the constant maintenance of motile cilia, and mutation in *FoxJ1* causes ciliopathy characterised by foetal hydrocephalus [47]. Hydrocephalus can also be induced through dysfunction of motile cilia on the ependymal cells by experimentally degrading the Foxj1 protein with I*κ*B kinase-2 inhibitors [52]. Furthermore, *Hydin*, a gene that encodes a central pair protein within the axon of motile cilia and expresses ependymal cell layer lining the ventricles, also may induce hydrocephalus when mutated [39, 40, 44]. *Mdnah5* is specifically expressed in ependymal cells, and is essential for ultra-structural and functional integrity of ependymal cilia [41]. Both the polycystin-1 encoded by the *Pkd1* gene, required for cilia function, and polaris encoded by the *Tg737* gene, required for cilia structure, are reported to be crucial mechanosensitive molecules to control fluid flow through primary cilia in endothelial cells [22, 53]. *Daple* regulates the direction and coordination of motile cilia beating in the ependymal cells, and determines the direction of CSF flow [54]. *Daple*-deficient mice present communicating hydrocephalus [54], and mutation in human *DAPLE* causes autosomal recessive congenital hydrocephalus [55]. Mutation in *DNAH14* results in motile cilia dysfunction was identified in familial cases diagnosed with panventriculomegaly with a wide foramen of Magendie and large cisterna magna (PaVM) that belongs to a subtype of late-onset congenital communicating hydrocephalus [43]. Mutation in *CFAP43*, encoding a protein associated with ependymal cilia, was also identified as a cause of familial NPH, and *CFAP43* knock-out mice exhibited hydrocephalus due to morphologic abnormality of motile cilia [42]. Two deletions in *CWH43* which regulates the membrane targeting of glycosylphosphatidylinositol-anchored proteins were identified in 8 of 53 iNPH patients (15 %), and *CWH43* mutant mice exhibited communicating hydrocephalus with gait and balance dysfunction [50]. Because *CWH43* mutant mice appear normal at birth and show no major deficits through middle age, they are considered to be the animal model close to iNPH or late-onset congenital or developmental hydrocephalus. The number of ependymal cilia of *CWH43* mutant mice was significantly decreased compared to that of wild type mice, although *CWH43* has not been reported to be a gene associated with dysfunction or degeneration of motile cilia and ependymal cells. This review excluded genes that were reported to be associated with cerebral aqueduct stenosis and non-communicating hydrocephalus. These genetic studies provide robust support regarding the importance of ciliary function and offer a commonality among the varied causes of communicating hydrocephalus. However, less is understood on how these gene mutations associated with ciliary dysfunction result in ventricle dilation. Other genes which were reported to cause hydrocephalus in rodents are associated with several inflammatory cytokines. Transgenic mice that overexpressed the transforming growth factor β1 (TGF-β1) die within 3 weeks after developing a severe form of hydrocephalus [56]. In the *hyh* mouse, one-third survive longer and exhibit congenital hydrocephalus [45, 57]. Here the cytokine tumour necrosis factor alpha (TNFα) and its receptor TNFαR1 were upregulated in the brain, particularly in the periventricular white matter lesion [57]. The production of TNFα in glial cells could mediate the permeability of the endothelium of the brain blood barrier [58]. Therefore, these inflammatory cytokines might affect CSF malabsorption due to dysfunction of the glymphatic pathway in the aetiology of iNPH.

### Environmental factors

Alcohol abuse during pregnancy is a known risk factor for foetal hydrocephalus [59, 60]. Furthermore, oral intake of ethanol was reported to decrease the beating frequency of motile cilia on the ependymal cells of lateral and third ventricles in a rat model [61]. Recently, two case-control studies provided evidence indicating that alcohol consumption showed a higher frequency in iNPH patients compared with an age-matched general population [62, 63]. Therefore, alcohol consumption is a potential risk factor for developing iNPH due to motile cilia dysfunction on the ependymal cells.

Patients with iNPH have a higher comorbidity rate of diabetes, which is more than double, compared with an age-adjusted general population [64–66]; however, the causal relationship between diabetes and iNPH has not yet been determined. Studies from patients with vestibular schwannoma have reported to have higher occurrence of communicating-type hydrocephalus, rather than non-communicating hydrocephalus due to the fourth ventricular compression [67–72]. Furthermore, sNPH related to vestibular schwannoma is frequently observed as focal dilation of the Sylvian fissure concurrent with ventricular enlargement, similar to DESH in iNPH [68, 72]. Following the complete removal of the schwannoma, 78–100% of sNPH patients were improved without persistent CSF shunt surgery [68–70]. Preoperative protein concentration in the lumber CSF was higher, [67] but significantly decreased after tumour removal [69]. Therefore, a higher protein concentration in CSF produced by schwannomas is a potential biomarker for communicating-type hydrocephalus pathogenesis associated with vestibular schwannoma. If sNPH associated with vestibular schwannoma is a reversible disease without CSF shunt surgery by tumor removal, permanent impaired absorption of CSF is unlikely to be the cause of hydrocephalus. Instead, dysfunction of CSF dynamics should be considered as the main cause of communicating-type hydrocephalus associated with vestibular schwannoma. A high concentration of sugars and proteins in CSF increases the fluid viscosity which in turn increases the shear stress on the ventricular wall surface, as shear stress is dependent on the flow velocity and fluid viscosity. *In vivo* experiments have shown that the increase in fluid viscosity enhances the dilatation of the vasculature, in parallel with changes in blood viscosity, through the sensitivity of the endothelium to shear stress [73, 74].

A higher frequency of sleep apnoea was reported in iNPH patients in a case-control study [75]. Indeed, there has been growing interest in the relationship between sleep and the glymphatic system [76–78]. The clearance activity of the glymphatic system is stimulated during sleep by increased CSF and interstitial fluid exchange. The amount of interstitial fluid increased by 60 % during sleep compared with being awake [78]. In addition, disorders of the sleep-wake cycle are closely associated with the accumulation of brain waste products, including amyloid-β and τ, and optimising the sleep-wake cycle would be important for the prevention of Alzheimer’s disease and other tauopathy [77, 79, 80]. Recently, deep non-rapid eye movements sleep has been reported to amplify reciprocating CSF movements synchronised with cerebral blood flow in conjunction with slow-delta electrophysiologic oscillations [81]. During deep sleep, the large slow waves of cerebral blood circulation and neural activity were interlinked to the CSF inflow into the ventricles through the foramen of Magendie.

In a nested case-control study, hypertension was reported to be a potential risk factor for hydrocephalic ventricular enlargement, which was distinct from ventricular enlargement in iNPH [65]. In rodents, angiotensin-II induced hypertension and decreased the speed of CSF net flow in the periarterial space by less than 40% [30], suggesting a decline in glymphatic function.

### Aging effect on ventricular enlargement in iNPH

Current CSF dynamic results suggest that age-related impairment of meningeal lymphatic CSF drainage and glymphatic fluid exchange between CSF and interstitial fluid may contribute to the pathogenesis of iNPH [32, 82–85]. Indeed, the meningeal lymphatic outflow of CSF is reduced in aged mice compared with young mice [86]. The reduction of cerebral blood flow, arterial pulsations and arterial elasticity could contribute to age-related glymphatic decline, and furthermore the age-related decline in sleep duration and efficiency may also impair the overall sleep-active glymphatic function. Sleep quality and quantity decline rapidly over the age of 65 and is characterised by the significant decrease of deep sleep with non-rapid eye movements-slow wave [80, 87 ]. In general, older individuals (> 65-years) sleep lighter and cannot stay asleep, resulting in awakening early in the morning. Because aging is considered to be the most relevant to the development of iNPH, the pathogenic mechanism of ventricular enlargement in iNPH may be associated with age-related sleep disorders. In addition, it is important to note the phenomenon of the aging brain, i.e. as the brain volume decreases, the intracranial CSF volume increases. Age-related declines in the function and number of motile cilia and ependymal cells also lead to ventricular enlargement in iNPH, as detailed in the next chapter.

### Oscillation of shear stress on the ventricular wall surface

**Fig. 3:**
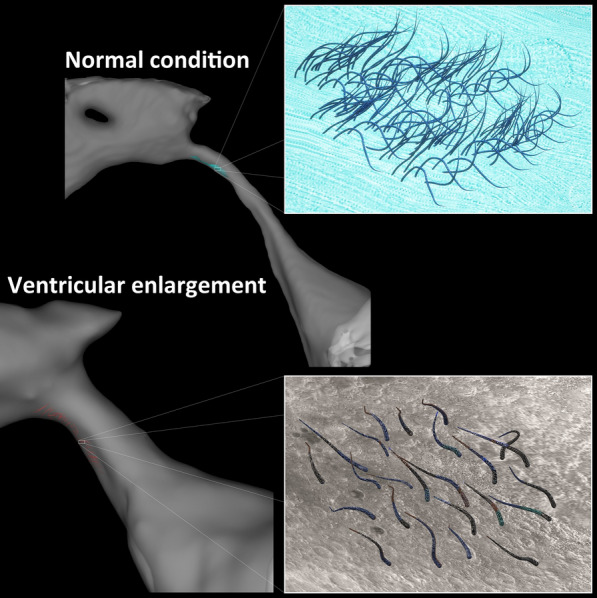
Illustration of cilia loss from the cerebral aqueduct wall surface due to oscillatory shear stress produced by the reciprocating CSF flow. This figure was drawn by our imagination using the Paint 3D application (Microsoft Corporation) and SYNAPSE 3D (FUJIFILM Corporation). The two upper figures depict the cerebral aqueduct in which the coordinated beating of motile cilia on the ependymal cells generate a directional steady flow of CSF under normal conditions. The lower figures illustrate the shedding of the motile cilia from the degenerated ependymal cells due to increased CSF backflow into the ventricles in iNPH

Under normal conditions, the movement of CSF in the cerebral aqueduct is much less than those in the foramen magnum and premedullary cistern [10, 17] The coordinated beating of motile cilia on the ependymal cells covering ventricular surface generates a directional steady laminar flow of CSF, which maintains the ventricles in a calm environment [20, 88]. Conversely, subarachnoid space is covered by the meningeal cells forming the leptomeninges, arachnoid and pia mater, and forms a transparent membrane composed of fibrous tissue with no motile cilia. The arachnoid membrane is connected to the pia mater by many trabeculae, just like a cobweb, and structurally continuous with the pia mater. In iNPH, the CSF stroke volume at the cerebral aqueduct is significantly increased, while unchanged at the foramen magnum [13, 17, 18]. Inevitably, the oscillatory shear stress produced by the reciprocating CSF flow acting tangentially on the wall surface is also increased in the cerebral aqueduct [18]. Increase in the CSF oscillation directly impedes normal cilia beating. The impaired beating of ependymal cilia is compounded by decreased apical actin filaments, which leads to motile cilia shedding from the ependymal cells, since the coordinated directional beating of motile cilia contributes to centriole stabilisation [88]. Oscillatory shear stress produced by the complex turbulent flow is known to be a detrimental cellular stress and be associated with autophagy in endothelial cells [89, 90]. The shedding of motor cilia can cause glial scarring on the surface of the ventricles by direct exposure to strong oscillatory shear stresses, as illustrated in Fig. 3. It has been demonstrated that the lateral ventricles expand due to the loss or degeneration of ependymal cell coverage on the ventricular surface [91].

Oscillation of the shear stress on the vascular endothelial cells has been noted in the pathogenesis of vascular dilation through activation of inflammatory signals [74, 92–97]. Compared with physiological steady shear stress, oscillatory shear stress induces higher activation of TGF-β1 which drives pathological vascular remodelling [94]. Endothelial cilia dysfunction contributes to aberrant fluid-sensing and thus results in vascular disorders, such as aneurysm formation and atherosclerosis [98, 99] Therefore, increased oscillatory shear stress due to reciprocating CSF movements may directly activate flow-mediated dilation at the cerebral aqueduct, and it may generate a synergistic effect of increased stroke volumes at the nearby upstream CSF pathways in the ventricles. Finally, the lateral ventricles might gradually expand due to an increase in oscillatory shear stress at the ependymal surface and loss of motile cilia and ependymal cells.

### Potential biomarker of cerebrospinal fluid indicating degeneration of motile cilia or ventriculomegaly

Recently, Nakajima et al. reported that iNPH patients had significantly higher concentration of the protein tyrosine phosphatase receptor type Q (PTPRQ) in the lumbar CSF (mean: 619 pg/mL), compared with either healthy control individuals (296 pg/mL), patients with Alzheimer’s disease (365 pg/mL) or Parkinson’s disease (338 pg/mL) [100]. Furthermore, the mean levels of PTPRQ for sNPH (1697 pg/mL) and late-onset congenital or developmental hydrocephalus (1720 pg/mL) were double that of iNPH levels. Patients with sNPH and late-onset congenital or developmental hydrocephalus usually had larger ventricles and higher CSF stroke volume at the cerebral aqueduct, compared with patients with iNPH, as depicted in Fig. 2. PTPRQ is a membrane protein involved in actin polymerisation and ciliary movement and structure. In addition, PTPRQ are expressed in the ependymal cells of the ventricles and choroid plexus indicated by immunostaining and *in situ* hybridisation. Therefore, PTPRQ concentration in the CSF is a good candidate as a CSF biomarker for ventricular enlargement in NPH due to the degeneration of motile cilia on the ependymal cells.

## Conclusions

**Fig. 4:**
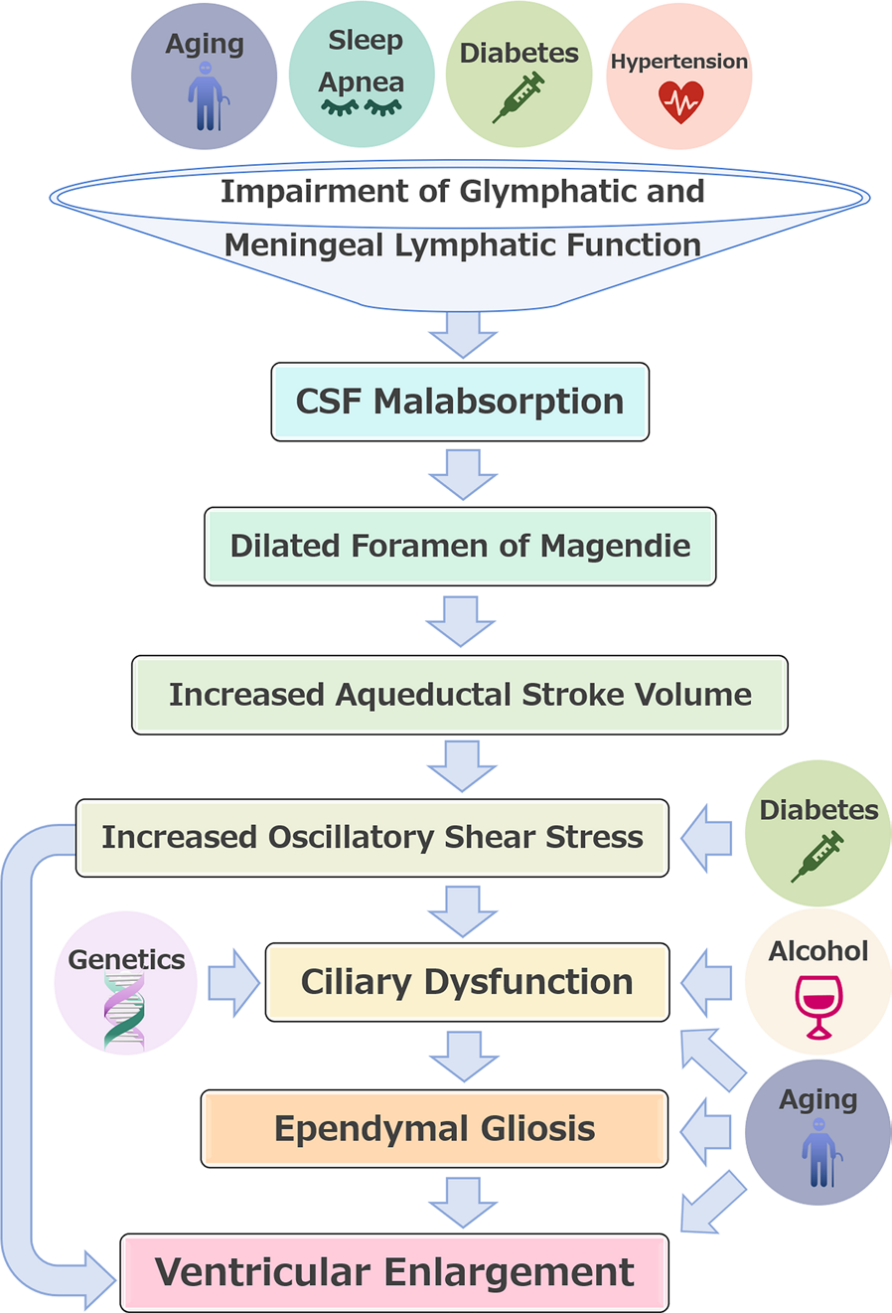
Potential mechanisms of ventricular enlargement in iNPH. The schematic diagram provides an overview of possible aetiological factors for ventricular dilatation in iNPH

This review summarises the current literature related to the mechanism of ventricular enlargement in iNPH. Although the aetiology of ventricular enlargement in iNPH has not yet been demonstrated, several reports suggest that the increase oscillatory shear stress on the ventricular wall, shedding of motor cilia and the subsequent loss of ependymal cell coverage may be crucially involved for ventricular enlargement in iNPH (Fig. 4). The cumulative evidence from these studies support the notion of CSF malabsorption as the combined impairment of glymphatic and meningeal lymphatic function is located upstream of ciliary dysfunction in the mechanism of ventricular enlargement in iNPH (Fig. 4). In summary, we hope that this review will be valuable for future basic and clinical research focusing on the mechanism of ventricular enlargement in iNPH.

## Data Availability

Not applicable.

## Abbreviations

CSF: Cerebrospinal fluid
DESH: Disproportionally enlarged subarachnoid space hydrocephalus
iNPH: Idiopathic normal pressure hydrocephalus
NPH: Normal pressure hydrocephalus
PaVM: Panventriculomegaly with a wide foramen of Magendie and large cisterna magna
PTPRQ: Protein tyrosine phosphatase receptor type Q
sNPH: Secondary normal pressure hydrocephalus
